# Screening for Intimate Partner Violence against Women in Healthcare Sweden: Prevalence and Determinants

**DOI:** 10.5402/2011/510692

**Published:** 2011-12-27

**Authors:** Stephen Lawoko, Sören Sanz, Lotti Helström, Maaret Castren

**Affiliations:** ^1^Division of Social Medicine, Department of Public Health Sciences, Karolinska Institutet, 17176 Stockholm, Sweden; ^2^Department of Emergency Medicine, Södersjukhuset, 18883 Stockholm, Sweden; ^3^Department of Clinical Science and Education, Karolinska Institutet, 17176 Stockholm, Sweden

## Abstract

We assessed the extent to which healthcare providers at a large healthcare facility in Sweden screen for intimate partner violence against women and the determinants of such screening. Data on frequency of screening, readiness to screen on many dimensions (using the Domestic Violence Healthcare Provider Survey Scale), demographic and occupational characteristics were administered electronically to 217 healthcare providers. We found that only 50% of participants had during the past 3 month screened for IPV at least once, and screening activity was marked with inequalities in measured individual characteristics. Participants of female gender and of doctor/nurse occupation were more likely to screen than male and midwife peers, respectively. Healthcare providers who perceived high efficacy in handling IPV issues, low fears of offending clients, professional preparedness, and with availability of support networks for IPV victims were more likely to screen for IPV. Implications of these findings for interventions are discussed.

## 1. Introduction

Intimate partner violence (IPV) is defined as behaviours within an intimate relationship that cause physical, sexual, or psychological harm, including acts of physical aggression, sexual coercion, psychological abuse, and controlling behaviours [1]. Though IPV is prevalent among both men and women, its impact on abused women's health is far more pronounced and documented, prompting call for action from reknown health researchers and organisations in a bid to break the cycle of abuse. Women experiencing IPV suffer a wide range of health complications resulting from physical, sexual, and psychological assaults manifest in severe physical injuries [2–4], reproductive health problems including terminated pregnancies, undesired pregnancies and child loss during infancy [5–7], symptoms of depression, anxiety and posttraumatic stress disorder, and risky health behaviours such as unhealthy feeding habits, substance abuse, alcoholism, and suicidal behaviours [8–10]. 

A disturbing aspect of intimate partner violence is the under usage of the healthcare system by abused women. Despite the higher likelihood for morbidity, women experiencing IPV when contrasted with nonabused peers use community and healthcare services disproportionately and exhibit a constricted contact with healthcare providers and employers when compared with peers in nonabusive intimate relationships [11, 12]. A recent WHO multicountry study of 10 countries including low, middle, and high income countries [11], found fear of retaliation from the abuser and stigmatizing attitudes from service providers and community at large to hinder abused women from seeking sanctuary from formal networks (e.g., healthcare). Instead, abused women opt for redress in such situations from informal networks (e.g., family and relatives) [13]. In Sweden, few abused women trust the judiciary system and only one in four is offered assistance from relevant formal institutions [14]. Overall, these findings could be a reflection of social and institutional marginalisation of abused women and/or the inability of formal institutions to assist IPV victims. The healthcare system thus could play an important role to reverse this notion through the institutional detection and management of IPV (i.e., screening for IPV in healthcare settings). 

In medicine, screening refers to a strategy used in a population to detect a disease in individuals with or without obvious signs or symptoms of that disease. In essence therefore, screening for IPV in healthcare is a systematic involvement of healthcare workers in the detection of IPV among clients who may or may not present with direct signs of victimization/abuse. The discussion on whether IPV screening should be universal is ongoing as the evidential support for the benefits of screening is little. However, self-reports from women indicating that they are comfortable responding to IPV-related inquiries in healthcare settings [15] together with recent evidence suggesting that such inquiries may reflect positively on women's satisfaction with healthcare in general [16] underscore the importance of universal screening. In support of this argument, healthcare professionals acknowledge that routine screening is likely to improve identification and management of IPV [17].

Despite consensus among stakeholders (i.e., women clients and healthcare practitioners) on the potential benefits of screening for IPV in healthcare, only about 8–10% of healthcare personnel routinely screen for the phenomena [18, 19], suggesting barriers. The healthcare providers' insufficient knowledge and training in screening could explain this discrepancy [18, 20]. Other factors related to professional roles governing the provider-client relations (e.g., mutual respect, fear of offending clients) and healthcare provider's individual attitudes towards IPV may influence screening for IPV in healthcare [21, 22]. An assessment of how structural factors inherent in healthcare supplier/supply (e.g., healthcare provider's skills and capabilities in screening, training, their attitudes towards IPV screening, and their access to support systems to which victims can be referred) may affect the likelihood of screening for IPV is warranted. Moreover, studies on possible demographic and occupational factors that may account for differences in the screening for IPV between individual healthcare providers are few in general [21–23] and lacking in the Swedish context. For example, it may be hypothesised that female care providers are more prone than male peers to inquire about IPV as they are more likely to identify with the problem, being potential victims; nurses may be more prone to inquire about IPV as they are more often at the forefront of care provision; experienced personale may be more likely to probe for IPV and so forth. An assessment of how such factors are related to screening is useful among other things for the identification of potential groups requiring further education in screening. Moreover, such baseline data will inevitably inform the development of training programs and screening protocols for IPV in healthcare Sweden. As of current, screening for IPV is voluntary practice in most Swedish healthcare organisations, as protocols for screening remain nonexistent. The current study will set the foundation for the introduction and development of routine screening in a healthcare facility in Sweden. 

 The main objective of this study therefore is to estimate the extent and predictors of screening in healthcare Sweden, using data from a large healthcare facility. Specifically, the following research questions will be tackled.

- To what extent do healthcare providers screen for IPV in Sweden?
- To what extent do work-related factors (i.e., availability of referrals for IPV victims, perceived efficacy in screening, conflicting professional roles), individual attitudes (e.g., blaming the victim), and occupation (e.g., being a doctor or nurse) influence the likelihood of screening for IPV?
- To what extent do demographic factors (e.g., sex, age, religion, being of ethnic majority/minority) influence the likelihood of IPV screening?

## 2. Methods

### 2.1. Study Settings, Design and Participants

The study was based on a cross-sectional survey design carried out at three departments (i.e., women's clinic, emergency clinic, and ambulatory department) of the Södersjukhuset, Sweden, one of the largest multidepartmental hospitals in the country. The healthcare providers at the hospital have not previously undergone any specific formal training in screening for IPV among their female clients. Thus, IPV screening remains undefined in routine practice. 

A total of 217 providers participated in the survey, though 77% of them responded to the question on screening for IPV (the outcome variable for this study). The effective sample for this study was thus 168 healthcare practitioners. None respondents did not however differ on demographic and occupational characteristics from those who responded.  Table 1 provides information regarding demographic and occupational distribution of the participants (effective sample). 

All employees at the above-mentioned departments were offered the opportunity to participate in this study through a web survey accessible by all employees (i.e., the hospital has an employee website accessible by all employees) The survey was available on the website during the period June–August of 2009. Information of the study was given by the respective department heads and further emphasised on the website. Voluntary participation was emphasised and informed consent was given. Participants were informed that responding to the questionnaire implied that they had consented to partake in the study.

### 2.2. Ethical Consideration

This study received ethical approval from the regional ethical review board. The aims and relevance of the study were explained to the participants and information on the same was offered on the web. Voluntary participation was emphasised, privacy guaranteed, and informed consent given. Participants' responses were anonymous.

### 2.3. Instrument Readaptation to Swedish Setting

The survey tool was first translated to the Swedish language, the official language in the country, by a professional translator. Another independent translator with good knowledge of both languages then translated the Swedish translation back to English. The authors, who master both languages, then studied and scrutinised the translation and agreed to adopt it with minor modifications. The team however also scrutinised the content of all 35 questions and their applicability in the studied setting. All questions relating to probing about IPV from the potential perpetrators (one of the subscales) were excluded from this study, the reason being that the organisation intends to introduce a screening protocol that exclusively probes of IPV possibility only among potential victims. There are no plans to probe of IPV issues from the perpetrators for safety reasons. The subscale was thus deemed irrelevant for the current sample and therefore excluded from the web survey.

### 2.4. Measures

#### 2.4.1. Dependent Variable

The dependent variable in this study was screening for IPV which was operationalized by probing of participants how often they had during the past 3 months inquired of the possibility of IPV from their female clients. IPV was defined for participants as exposure to physical, sexual, or psychological abuse. Examples of these forms of abuse were given. Because the distribution of this variable was grossly skewed to the left (i.e., about half of the participants had not inquired of IPV at all during this period) a dichotomous variable was computed for further analysis of predictors of screening as follows: 1 if screened for IPV at least once, 0 otherwise.

#### 2.4.2. Independent Variables

The *Domestic Violence HealthCare Provider Survey Scale* (DVHSS) [21] was used to measure work-related factors hypothesised to influence screening behaviour. The instrument has previously been validated with promising results in some countries including USA and Nigeria [21, 24]. The questionnaire, in its original format, is composed of the following 5 subscales, though only 4 of the subscales were used in this study. These are the following.

- The *perceived self-efficacy subscale (4 items)* which assesses healthcare providers' own perceived efficacy in inquiring about IPV from potential victims in terms of having strategies for screening, time to screen, confidence to make referrals for victims, and access to information on IPV management. Cronbach's alpha testing for internal consistency for the current sample was 0.74, indicating good internal consistency.
- The *system support subscale (4 items)* assessing healthcare providers' access to support from mental health workers, social assistants, and community advocates. Cronbach's alpha testing for internal consistency for the current sample was 0.68, indicating good internal consistency.
- The *professional roles resistant/fear of offending clients subscale (7 items) *assesses whether providers perceive inquiries about IPV to conflict with professional conduct governing their communication with clients. Specifically, participants are probed on whether IPV inquiry in their view is an invasion of patient privacy, demeaning to patient, may provoke anger, offensive, not part of medical practice, none of their business. Cronbach's alpha testing for internal consistency for the current sample was 0.68, indicating good internal consistency.
- The *blame victim subscale (8 items)* assesses providers' attitudes towards victims. Generally, participants are probed on whether they feel victims are getting something out of the abuse, choose to be victims or deserve abuse, and whether victims personalities and breaking of social norms are the cause of abuse. Cronbach's alpha testing for internal consistency for the current sample was 0.76, indicating good internal consistency.

Individual scores for each of the above scales range from 1–5.

*Demographic variables* included as independent variables in this study were age, sex, minority status, marital status and religion, while *occupational characteristics* included department, employment category, and years of experience.

### 2.5. Statistical Analysis

#### 2.5.1. Data Cleansing

Prior to analyses, certain procedures were carried out to clean data. First, only participants who had responded to the question on IPV screening (the dependent variable) were included. Second, Cronbach's Alpha testing for internal consistency of the subscales of the DVHSS for the current sample was calculated. Third, the DVHSS subscales were scored in accordance with previous recommendations (i.e., individual means on each scale) [21] and the resulting means checked for normality using the skewness statistic and its confidence interval. Skewness statistic of magnitude zero is an indication of perfect symmetry. Individual means on the subscales followed a normal distribution, thus conventional statistical methodology assuming normality described below could be adopted.

#### 2.5.2. Data Analysis

To respond to the research questions, a number of statistical analyses were run. *T*-tests were used to assess whether scores on the DVHSS differed between participants who had screened and those who had not. To study the association between continuous demographic/occupational measures (i.e., age and working experience) and the dichotomous outcome variable (i.e., screening or not) *T*-tests were run. To study the association between categorical demographic/occupational measures (e.g., sex and employment categories) and the dichotomous outcome variable (i.e., screening or not) Chi-square tests were run. Finally, to assess the independent association between dependent and independent variables logistic regressions analysis was run. For all tests, statistical significance was assumed at *P* < 0.05. Statistical Package for Social Sciences (SPSS version 19.0) was used for all analyses.

## 3. Results

### 3.1. Extent of Screening and Bivariate Associations with Demographic/Occupational Factors

Of the 168 participants, 51%  (*n* = 86) had during the past 3 months screened for the possibility of IPV among their female clients at least once. As shown in Table 2, the likelihood of screening for IPV at least once during the past month varied depending on certain demographic/occupational characteristics. Female participants screened to a higher extent than male peers (*P* < 0.05) and midwives to a lower degree than other staff cadres (*P* < 0.05). The differences in screening observed by marital status, migrant status, department, age, and years in service did not reach statistical significance.

### 3.2. Bivariate Associations between Screening and Enabling Work Factors

As indicated in Table 3, participants who screened for IPV when contrasted with peers who did not perceived on average a higher self-efficacy (*P* < 0.001), higher availability of a support network for IPV (*P* < 0.01), and lower conflicting professional roles and fears with regard to IPV screening (*P* < 0.05). The hypothesised association between screening and victim blame did not reach statistical significance.

### 3.3. Independent Predictors of Screening for IPV

Variables that were significantly associated with IPV screening in the bivariate analyses were considered as candidate variables for multivariable analyses to assess their independent association with the likelihood of screening. However, some of these variables posed a risk for multicollinearity (i.e., high correlation between self-efficacy, system support, and professional role resistant/fear of offence) and could not therefore be included in the same model. Thus, three different regression models were run to avoid the problem as presented in Table 4.

As indicated in Table 4, gender remained consistently associated with screening even after control for other factors (i.e., staff cadre, self-efficacy, professional role resistance, and system support). Male participants were consistently less likely to screen than female peers, as expressed by the odds ratios and their confidence intervals. In models 2 and 3, midwives exhibited a lower likelihood of screening than did doctors even after control for other factors. After controlling for gender and staff cadres, the likelihood of screening for IPV increased with increasing self-efficacy (model 1), reducing professional role conflicts/fears (model 2), and increasing availability of support networks (model 3).

The three models explained 25%, 18%, and 16%, respectively, of the variation in IPV screening.

## 4. Discussion

We examined the extent and predictors of IPV screening at a large health facility in Sweden using a cross-sectional study design. About 50% of the participants reported having inquired about the possibility of IPV from their female clients at least once during the past three months. These figures appear higher than what is reported in Kano Nigeria [22], a low income setting, but still does not meet the ideal level expected of screening activity. According to advocates for IPV screening [1, 22], the practice should ideally be both routine and universal, that is, personale should inquire of the possibility of IPV of all their female clients routinely, regardless of whether they show clear symptoms of abuse or not. Following this strict protocol, one would have expected to see much higher figures to assume routine screening. The findings therefore call for systematic training in IPV screening at the study context (i.e., Södersjukhuset) and similar health clinics in Sweden, if ideal goals for IPV screening are to be met. 

Screening activity was significantly associated with demographic and occupational factors. Female healthcare professionals were more likely to screen for IPV than male colleagues, corresponding with hypotheses suggesting that women may be more prone to screen for IPV out of sympathy for fellow women. The findings could also be echoing previous work indicating that women patients may be more prone to discuss IPV issues with female professionals [25]. Doctors and nurses appeared better placed than midwives to inquire of IPV exposure. These findings signal inequalities in readiness to screen with regard to profession and call for a review of prevailing protocols on how to handle patients, how such protocols differ across different staff cadres, and what influences such difference could have on IPV screening activity. As is previously recommended [26–28], there is a need for formulation of clear guidelines on IPV screening for clinicians. Such guidelines should however be sensitive to the differences in professional roles that may deter/foster screening activity.

Not surprisingly, healthcare providers who perceived high efficacy in handling IPV issues, low fears of offending clients, professional preparedness, and availability of support networks (all with regard to IPV screening) were more likely to screen for IPV. These findings corroborate work from other settings [21, 22] and stress the need to tailor training programmes to address fears and possible role conflicts when confronted with IPV-related inquiries. In addition, structural improvements both within and/or outside the healthcare setting are warranted to improve healthcare providers' knowledge of the support networks available for them to refer IPV victims. This could be achieved through further education, provision of elaborate information on existing relevant organisations, and improved collaboration with community and other relevant services that address abuse of women. 

Though the study corroborates some previous works and provides direction for the programming of IPV screening in Sweden, some of its weaknesses deserve some acknowledgement. First, the issue of selection bias cannot be totally ruled out. It is difficult to compare the characteristics of those who responded to the web survey with those who did not. One could question whether respondents were more likely to be sympathetic to the subject at hand. If that was the case, then our results concerning the extent of screening could be an underestimate. Likewise, it is possible that only the computer literate may have participated. Nonetheless, Sweden is a highly computer-literate society and this to some extent diffuses suspicions of any biases introduced by variations in computer literacy in the study population, particularly given that they comprise professionals predominantly. With the same token, it is not known whether any differences that may be significant for screening are likely between those who choose to work in the summer and those who do not, as data was collected in the summer season. Finally, because of its cross-sectional design the study can only confirm associations but cannot draw casual inference. Notwithstanding, these plausible weaknesses many findings are in line with other works suggesting inequalities in screening activity due to demographic, occupational, work-environmental, and structural factors [18–25].

## Figures and Tables

**Table 1:** Demographic and occupational characteristics of participants.

	*N*	*%*
Gender		
Male	36	21
Female	132	79
Immigration status		
Immigrant*	24	14
Swedish born	144	86
Marital status		
Married/cohabiting	116	69
Single	35	21
Divorced/widower/widow	17	10
Staff cadre**		
Doctor	25	16
Nurse	62	40
Nursing assistant	34	22
Midwife	26	17
Other	7	5
Department**		
Emergency	106	65
Women's clinic	45	28
Ambulatory	12	7
Age		
(mean (st.dev in brackets))	41 years (10.0 years)	
Years in service		
(mean (st.dev in brackets))	17 years (11.2 years)	

*N*: number within category, %: proportion within category. *Born of another nationality than Swedish. **Not all participants responded to these inquiries. Thus total falls short of *n* = 168.

**Table 2:** Screening for IPV by demographic and occupational characteristics of participants.

	Number screening (*n*)	Proportion *% *
Gender*		
Male	12	34
Female	70	53
Immigration status		
Immigrant	10	43
Swedish born	72	50
Marital status		
Married/cohabiting	57	49
Single	15	43
Divorced/widower/widow	10	58
Staff cadre*		
Doctor	14	56
Nurse	31	50
Nursing assistant	20	59
Midwife	7	27
Other	6	85
Department		
Emergency	58	55
Women's clinic	16	35
Ambulatory	4	33

	Screened Mean (SE)	Did not screen Mean (SE)

Age (years)	41 (1.1)	40 (1.1)
Years in service	17 (1.2)	18 (1.3)

*n*: number within category that screened, %: proportion within category that screened. S.E: Standard Error. *Denotes statistically significant association between screening and respective variable at *P* < 0.05.

**Table 3:** Association between screening and DVHSS subscales.

	Screened for IPV Mean (SE)	Did not screen for IPV Mean (SE)
Perceived self-efficacy***	3.79 (0.09)	3.04 (0.09)
System support**	3.52 (0.09)	3.10 (0.10)
Blame victim	1.67 (0.06)	1.57 (0.06)
Professional role resistant/fear of offence*	1.53 (0.05)	1.70 (0.06)

*Denotes statistically significant at *P* < 0.05; **denotes statistically significant at *P* < 0.01; ***denotes statistically significant at *P* < 0.001.

**Table 4:** Logistic regression showing predictors of IPV screening.

	Model 1 odds-ratio (CI)	Model 2 odds-ratio (CI)	Model 3 odds-ratio (CI)
Gender			
Female	1.00	1.00	1.00
Male	0.31 (0.11–0.84)*	0.32 (0.11–0.87)*	0.28 (0.10–0.77)*
Staff cadre			
Doctor	1.00	1.00	1.00
Nurse	0.60 (0.21–1.75)	0.65 (0.23–1.83)	0.55 (0.20–1.55)
Nursing assistant	0.75 (0.23–2.45)	0.81 (0.26–2.49)	0.79 (0.25–2.45)
Midwife	0.38 (0.09–1.53)	0.16 (0.04–0.60)*	0.21 (0.06–0.80)*
Other	4.18 (0.38–45.51)	5.67 (0.53–59.83)	5.49 (0.52–57.18)
Self-efficacy	2.33 (1.44–3.76)*	NA	NA
Professional role/fear	NA	0.49 (0.25–0.97)*	NA
System support	NA	NA	1.33 (1.01–2.21)*
Model *R*-square	*0.25*	*0.18*	*0.16*

CI denotes confidence interval for odds ratio. CI excluding 1 is an indication of statistical significance. NA: not included in model. *Denotes statistically significant at *P* < 0.05.

## References

[1] World Health Organisation (1997). Violence against women: a priority health issue.

[2] Aimakhu CO, Olayemi O, Iwe CAB (2004). Current causes and management of violence against women in Nigeria. *Journal of Obstetrics and Gynaecology*.

[3] Koenig MA, Ahmed S, Hossain MB, Alam Mozumder ABMK (2003). Women’s status and domestic violence in rural Bangladesh: individual- and community-level effects. *Demography*.

[4] Fawole OI, Aderonmu AL, Fawole AO (2005). Intimate partner abuse: wife beating among civil servants in Ibadan, Nigeria. *African Journal of Reproductive Health*.

[5] Garcia-Morena C, Jansen H, Ellsberg M, Heise L, Watts C (2005). WHO Multi-country study on women’s health and domestic violence against women: initial results prevalence, health outcomes and women’s responses.

[6] Kishor S, Johnson K Profiling violence: a multi-country study.

[7] Koss MP (1990). The women’s mental health research agenda: violence against women. *American Psychologist*.

[8] Golding JM (1999). Intimate partner violence as a risk factor for mental disorders: a meta-analysis. *Journal of Family Violence*.

[9] Heise L, Garcia-Moreno C, Krug E, Dahlberg LL, Mercy JA (2002). Violence by intimate partners. *World Report on Violence and Health*.

[10] Tjaden P, Thoennes N (2000). Extent, nature, and consequences of intimate partner violence: findings from the national violence against women survey.

[11] Tolman RM, Rosen D (2001). Domestic violence in the lives of women receiving welfare: mental health, substance dependence, and economic well-being. *Violence Against Women*.

[12] Petersen R, Gazmararian J, Andersen Clark K (2001). Partner violence: implications for health and community settings. *Women’s Health Issues*.

[13] World Health Organisation (2005). WHO multi-country study on women’s health and domestic violence against women.

[14] Brottsförebygande Rådet (BRÅ) (2009). Våld mot kvinnor och män i nära relationer: våldets karaktär och offrets erfarenhet av kontakter och rättsväsendet. *BRÅ rapport*.

[15] Stenson K, Sidenvall B, Heimer G (2005). Midwives’ experiences of routine antenatal questioning relating to men’s violence against women. *Midwifery*.

[16] John IA, Lawoko S, Oluwatosin A (2011). Acceptance of screening for Intimate Partner Violence, actual screening and satisfaction with care amongst female clients visiting a health facility in Kano, Nigeria. *African Journal of Primary Health Care & Family Medicine*.

[17] Furniss K, McCaffrey M, Parnell V, Rovi S (2007). Nurses and barriers to screening for intimate partner violence. *MCN The American Journal of Maternal/Child Nursing*.

[18] Erikson MJ, Hill TD, Siegal RM (2001). Barriers to domestic violence screening in the padiatric Setting. *Pediatrics*.

[19] Roelens K, Verstraelen H, Van Egmond K, Temmerman M (2006). A knowledge, attitudes, and practice survey among obstetrician- gynaecologists on intimate partner violence in Flanders, Belgium. *BMC Public Health*.

[20] Waalen J, Goodwin MM, Spitz AM, Petersen R, Saltzman LE (2000). Screening for intimate partner violence by health care providers: barriers and interventions. *American Journal of Preventive Medicine*.

[21] Maiuro RD, Vitaliano PP, Sugg NK, Thompson DC, Rivara FP, Thompson RS (2000). Development of a health care provider survey for domestic violence: psychometric properties. *American Journal of Preventive Medicine*.

[22] John IA, Lawoko S, Svanström L (2011). Screening for IPV in healthcare in Kano, Nigeria: extent and determinants. *Journal of Family Violence*.

[23] John IA, Lawoko S, Svanström L, Mohammed AZ (2010). Health care providers’ readiness to screen for intimate partner violence in northern Nigeria. *Violence and Victims*.

[24] John IA, Lawoko S (2010). Assessment of the structural validity of the domestic violence healthcare providers survey questionnaire. *Journal of Injury & Violence Research*.

[25] Thackeray J, Stelzner S, Downs SM, Miller C (2007). Screening for intimate partner violence: the impact of screener and screening environment on victim comfort. *Journal of Interpersonal Violence*.

[26] Caden AD (1994). Wife abuse and the wife abuser: review and recommendations. *Counseling Psychologist*.

[27] World Health Organization (2002). The world report on violence and health.

[28] American College of Gynocologist and Obstetricians (ACOG) and Center for Disease Control (CDC) Intimate Partner Violence during pregnancy: a guide for clinicians.

